# Zero echo time MRI with deep learning reconstruction and chemical shift correction for detecting osteolytic myeloma lesions

**DOI:** 10.1186/s41747-026-00734-x

**Published:** 2026-06-05

**Authors:** Darius Lepot, Caroline Chabot, Gaëtan Duchêne, Sagar Mandava, Maggie Fung, Julie Poujol, Marie-Christiane Vekemans, Perrine Triqueneaux, Nina Watté, Olivier Gheysens, Nicolas Michoux, Frédéric E. Lecouvet

**Affiliations:** 1 Department of Medical Imaging, Institut de Recherche Expérimentale et Clinique (IREC), Institut du Cancer Roi Albert II, Cliniques Universitaires Saint Luc, Université catholique de Louvain (UCLouvain), Brussels, Belgium; 2 GE HealthCare MR Clinical Solutions, Atlanta, GA USA; 3 GE HealthCare MR Clinical Solutions, New York, NY USA; 4 GE HealthCare, Buc, France; 5 Department of Hematology, Institut de Recherche Expérimentale et Clinique (IREC), Institut du Cancer Roi Albert II, Cliniques Universitaires Saint Luc, Université catholique de Louvain (UCLouvain), Brussels, Belgium; 6 Department of Nuclear Medicine, Institut de Recherche Expérimentale et Clinique (IREC), Institut du Cancer Roi Albert II, Cliniques Universitaires Saint Luc, Université catholique de Louvain (UCLouvain), Brussels, Belgium

**Keywords:** Artificial intelligence, Bone, Deep learning, Magnetic resonance imaging, Multiple myeloma

## Abstract

**Objective:**

We compared three magnetic resonance imaging (MRI) sequences—native zero echo time (ZTE), deep learning (DL)-chemical shift correction (CSC) reconstructed ZTE (ZTE-DLCSC), and gradient-echo black bone (BB)—in detecting osteolytic multiple myeloma (MM) lesions, using computed tomography (CT) as reference.

**Materials and methods:**

Patients who had undergone whole-body ^18^F-fluorodeoxiglucose-positron emission tomography/CT were prospectively enrolled at a single-center and underwent 3-T whole-body MRI with ZTE and BB sequences covering the lumbar spine, pelvis, and proximal femurs. ZTE-DLCSC images were reconstructed from raw data. Ten bone regions were assessed for lesion presence and number by a senior radiologist with 26 years’ experience and two junior readers (a radiology fellow and a resident). Repeatability and reproducibility (Gwet’s agreement coefficients AC1 and AC2), differences in quantitative counts, and accuracy were evaluated *per*-sequence/region/reader.

**Results:**

Ten patients, aged 67 ± 12 years (mean ± standard deviation), were enrolled. Considering all regions, repeatability was at least moderate for ZTE (AC1 ≥ 0.45), good for ZTE-DLCSC and BB (AC1 ≥ 0.60) and very good for CT (AC1 ≥ 0.80). Reproducibility was at least fair for ZTE and BB (AC2 ≥ 0.20), good for ZTE-DLCSC (AC2 ≥ 0.60) and very good for CT (AC2 ≥ 0.80). Accuracy of ZTE-DLCSC ranged 80‒93%; compared to ZTE, accuracy increased by 23% and 25% for junior readers (*p* = 0.010 and *p* = 0.002 respectively) and by 32% for the senior reader (*p* < 0.001). ZTE-DLCSC detected more lesions than ZTE/BB (+30%, *p* = 0.011; +25%, *p* = 0.024).

**Conclusion:**

DL-driven DLCSC reconstruction improves the reliability and accuracy of the ZTE sequence for detecting MM lesions.

**Relevance statement:**

The combination of deep learning reconstruction and chemical shift correction improves the accuracy of zero echo time MRI for detecting myeloma lesions.

**Trial registration:**

This study was approved by the institutional ethics committee and registered at ClinicalTrials.gov (NCT05381077).

**Key Points:**

- The performance of ZTE-DLCSC for detecting osteolytic MM lesions was compared with that of native ZTE and BB sequences using CT as reference.
- ZTE-DLCSC showed better intra-reader agreement (repeatability) and inter-reader agreement (reproducibility) than ZTE and BB sequences.
- ZTE-DLCSC had fewer false positives and false negatives than ZTE and BB sequences.
- ZTE-DLCSC detected more lesions than ZTE and BB sequences.

**Graphical Abstract:**

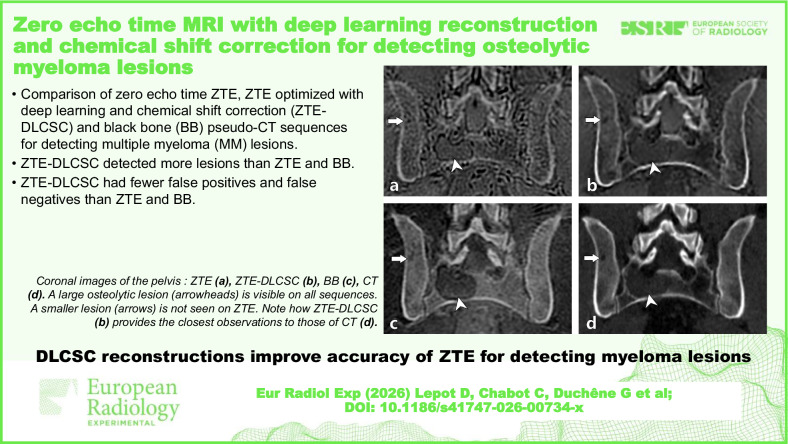

## Background

Whole-body magnetic resonance imaging (MRI) using morphological and diffusion-weighted imaging sequences is the method of choice for assessing bone marrow involvement in multiple myeloma (MM). It enables detection of marrow infiltration, evaluation of treatment response through diffusion-weighted imaging, and assessment of fat replacement. However, conventional MRI has long been limited by its inability to visualize cortical bone and detect osteolysis [1–3]. Computed tomography (CT), with its high sensitivity for osteolysis and high spatial resolution, is considered the method of choice for detecting lytic MM foci [4]. Recent advances in MRI have aimed to bridge this gap. Pseudo-CT MRI sequences now offer the potential to visualize the mineral structure of bone [5]. Recently developed zero echo time (ZTE) and black bone (BB) sequences allow complementing bone marrow exploration with mineral bone assessment, eliminating the need for additional ionizing examinations [6–8]. They have been evaluated in several clinical applications, including assessing trauma and degenerative or inflammatory joint conditions, detecting calcified deposits, and characterizing osteolysis and sclerosis in bone tumors [9–14].

In MM, pseudo-CT MRI sequences can complement anatomical sequences to evaluate the bone marrow by providing information on osteolysis that was previously evaluated using CT [15, 16]. A recent study in MM patients showed that ZTE was slightly less effective than BB sequences for detecting lytic lesions due to limitations such as a low signal-to-noise ratio and chemical shift (CS) artifacts caused by the proximity of fatty and non-fatty marrow contents. In this study, sensitivity ranged from 40% to 94% for ZTE and from 75% to 100% for BB. The specificity was between 73% and 100% for both ZTE and BB [16].

Solutions have been developed to overcome these limitations of the ZTE sequence. Deep learning (DL) reconstruction algorithms enable image denoising, significantly improving the quality and resolution of standard MRI sequences and offering faster acquisition [17, 18]. The role of DL reconstruction in improving the diagnostic value of the ZTE sequence for studying inflammatory involvement of the sacroiliac joints has been demonstrated [19]. Another study used an algorithm combining DL reconstruction and CS correction (CSC) to reduce CS artifacts and enhance signal-to-noise when evaluating temporomandibular joint osteoarthritis [20].

The CS between fat and water causes phase accrual in the fat signal during ZTE imaging. This phase accrual leads to blurring or destructive interference at fat–water interfaces, which degrades image quality [21, 22].

To mitigate CS artifacts, a prototype ZTE sequence combining DL reconstruction and CSC (ZTE-DLCSC) was built to reconstruct raw data. The aim of our study was to assess the value of this sequence combining DL reconstruction and CSC to address the limitations of the native ZTE sequence in MM, *i.e*., to bridge the residual gap for an optimal detection of osteolysis with this ZTE sequence. Therefore, we compared the ZTE-DLCSC sequence to native ZTE and BB sequences in a prospective series of MM patients, using CT as the reference standard. We hypothesized that DL and CSC would improve the diagnostic accuracy of ZTE in the detection of lytic MM lesions.

## Methods

### Study design and participants

This prospective, proof-of-concept single-center study was approved by the institutional ethics committee (2020/27JUL/380) and is registered on ClinicalTrials.gov (NCT05381077). Written informed consent was obtained from all participants. Patients were enrolled between February and July 2023. The inclusion criteria were patients with newly diagnosed MM who were referred by the hematology department to the nuclear medicine department for an ^18^F-fluorodeoxyglucose (FDG)-positron emission tomography (PET)/CT and who also underwent a supplemental whole-body MRI examination that included pseudo-CT sequences as part of the current study. The sample size was chosen for exploratory purposes, to compare the different pseudo-CT sequences on a limited skeletal area, and select the most effective one for a later phase of the project covering the whole body. The exclusion criteria were standard contraindications for MRI, *i.e*., incompatible medical devices, intraocular metallic foreign bodies and severe claustrophobia.

### Context

GE HealthCare supported this study by assisting with the implementation and optimization of ZTE and BB MRI sequences and by providing the DL-CSC algorithm. Authors (S.M., M.F., J.P.) are GE HealthCare employees who provided this support. These authors had no control over the data or analysis in the study. The other authors are not GE HealthCare employees and controlled the inclusion of all data and information that might present a conflict of interest for the GE HealthCare employees.

### Image acquisition

All MRI studies were performed on the same 3-T unit (SIGNA™ Premier; GE HealthCare). The protocol covered the whole body in multiple stacks from the vertex to the knees, including a three-dimensional (3D) T1-weighted fast spin-echo sequence, a 3D Dixon T1-weighted sequence (in/out phase, fat only, water only and fat fraction maps) and a diffusion-weighted sequence from which apparent diffusion coefficient maps were reconstructed (Table 1). In addition, 3D ZTE and BB sequences were acquired of the lumbar spine, pelvis, and proximal femurs, regions that are predominantly affected by osteolytic lesions in MM [23]. Detailed information on these sequences can be found in the literature [24–26]. The CT images were part of a standard ^18^F-FDG-PET/CT (Vereos; Philips Healthcare), and their reconstruction was optimized using dedicated parameters for studying the mineral bone, as previously described in the literature [16].

**Table 1:** Acquisition parameters for the whole-body MRI and pseudo-CT sequences (BB, ZTE and ZTE-DLCSC)

Sequence	FSE T1-weighted	DWI	IDEAL-IQ	BB	ZTE	ZTE-DLCSC
Mode and plane of acquisition	3D Coronal	2D Axial	3D Axial	3D Axial	3D Coronal	id. ZTE
Field of view (cm)	40 × 36 × 25.9	50 × 37.5 × 20	44 × 35.2 × 20.4	50 × 40 × 26.5	44 × 44 × 29	id. ZTE
Voxel size (mm)	1 × 1.5 × 1.8	4.1 × 4.1 × 5.0	2.9 × 2.9 × 3	1.6 × 1.6 × 1.6	1.6 × 1.6 × 1.6	id. ZTE
TR/TE (ms)	734/14	3,581*/59	5.6/0.8, 1.48, 2.16, 2.84, 3.52, 4.2	4.2/1.1, 2.4	–/0	id. ZTE
Flip angle (°)	90	90	3	2	1	id. ZTE
Number of excitations	1	1 (*b*-value = 50 s/mm^2^), or 2 (*b*-value = 800 s/mm^2^)	1	2	2	id. ZTE
Breathing management	Free breathing	Respiratory gating for chest and abdomen	Single breath hold for chest and abdomen	Free breathing	Free breathing	id. ZTE
Number of slices	288	40	68	332	360	Id. ZTE
Bandwidth (kHz)	62.50	250	111.11	142.86	62.50	Id. ZTE
Acquisition time per stack	3:20 min:s	1:17 min:s	20 s	2:16 min:s	2:50 min:s	/
Number of stacks	4	6	7	1	1	id. ZTE

Participants were positioned supine. Two 30-channel anterior air coils combined with a 40-channel table coil and a 21-channel head-and-neck coil were used to receive signals; a belt was installed on the abdomen for the sequences needing respiratory gating. Depending on the patient’s respiratory rate and breath-hold capability, the examination duration ranged 55–65 min. IDEAL-IQ is a chemical shift-encoded fat quantification approach. BB with a low FA is a 3D T1 liver acquisition with volume acceleration (LAVA) sequence acquired with the Dixon method. The ZTE is a 3D radial acquisition sequence with ultrafast signal readout

*2D* Two-dimensional, *BB* Black Bone, *DWI* Diffusion-weighted imaging, *FA* Flip angle, *FSE* Fast spin echo, *Id. ZE* Parameters identical to those used for ZTE, *IDEAL-IQ* Iterative decomposition of water and fat with echo asymmetry and least squares estimation, *TE* Echo time, *3D* Three-dimensional, *TR* Repetition time, *ZTE* Zero echo time, *ZTE-DLCSC* ZTE sequence reconstructed with deep learning and chemical shift correction algorithm

* When respiratory gating was used, TR was determined by the patient’s respiratory rate. The acquisition time was adjusted accordingly

### ZTE sequence optimization

The CS between fat and water leads to a phase accrual in the fat signal over the course of the imaging readout in ZTE. This accrued phase manifests as blurring or destructive interference at fat–water tissue interfaces and is a source of image quality degradation. The CS artifact is mitigated by the ZTE-DLCSC sequence in the form of reconstruction of the raw data of the sequence by using the algorithm combining “AIR™ Recon DL” and CSC (a vendor-supplied prototype of this reconstruction algorithm, specifically designed for ZTE was used to reconstruct the acquisitions offline). The ZTE AIR™ Recon DL prototype consists of a feed-forward deep convolutional neural network embedded in the image reconstruction path that generates enhanced images from complex-valued raw data. The network is trained *via* a supervised learning approach using more than 10,000 labeled image pairs and seeks to reduce noise, truncation and CS artifacts [27].

### Image analysis

All images (ZTE, ZTE-DLCSC, BB, CT) were evaluated on PACS workstations (Carestream Vue; Philips) using the windowing, multiplanar reformation, linking and scrolling tools. A gray-scale inversion was applied on pseudo-CT sequences to allow readings in similar windows as the CT, where bone appears white. All readings were randomized and independent, and were blinded to patient clinical data, other imaging examinations, and other MRI sequences.

A senior radiologist (R1) with 26 years’ experience in onco-hematological MRI and CT performed the readings once. To assess intra-reader repeatability, a radiology fellow (R2) with 6 years’ experience performed the readings twice, 4 months apart, to avoid recall bias. A radiology resident (R3) with 4 years’ experience performed the readings once. The pseudo-CT readings (ZTE, ZTE-DLCSC and BB) were performed first, at a 1-month interval between each sequence type. Following completion of readings of all pseudo-CT MRI sequences, each reader individually performed the CT readings at least 1-month later. Finally, all readers performed a consensus reading of the CT, blinded to the pseudo-CT MRI sequences, and this reading was considered the reference standard to detect osteolytic foci.

Ten bones (L1, L2, L3, L4, L5 vertebrae; sacrum; right and left coxal bones; right-left proximal femurs) were studied. In each bone, two prespecified scores were recorded: a categorical score (osteolytic MM lesion present/absent: score = 1 or 0) and a quantitative score (number of MM lesions).

A focal osteolytic MM lesion was defined as a focal area of trabecular bone destruction, with a diameter ≥ 5 mm, without a sclerotic border, contrasting on low-dose CT and pseudo-CT images with the well-mineralized adjacent trabecular bone [28, 29].

The following non-MM lesions were also noted [30]: vertebral hemangiomas, as focal lucent areas with residual internal thickened vertical trabeculae producing a “polka-dot sign” on axial images and “corduroy” on sagittal images, with a frequent fat content; Schmorl nodes, as radiolucent areas adjacent to a vertebral endplate resulting from herniation of nucleus pulposus within the vertebral body; subchondral cyst or geodes, as well-defined radiolucent areas abutting an articular surface; bone islands, or enostoses, as small areas of compact bone within cancellous bone with a density similar to that of cortical bone, blending imperceptibly with the surrounding trabeculae.

### Statistical analysis

Summary characteristics of the patient population are presented as means and frequency counts.

Repeatability of the readings (*i.e*., intra-reader agreement) for lesion detection (categorical score) was assessed from the 1st and 2nd reading from R2 using Gwet’s agreement coefficient 1 (AC1) [31]. Reproducibility of the readings (*i.e*., inter-reader agreement) was assessed from the 1st reading from all readers using Gwet’s agreement coefficient 2 (AC2). The strength of agreement was interpreted as follows: < 0.20, poor; ≥ 0.20 but < 0.40, fair; ≥ 0.40 but < 0.60, moderate; ≥ 0.60 but < 0.80, good; ≥ 0.80, very good. These analyses were performed *per*-sequence and *per*-region.

As ten bone regions *per*-patient were analyzed, a Goodman-Kruskal gamma for ordinal data was first computed to test the independence between regions and patients, in terms of “error” using MRI compared to the consensus CT for lesion detection [32]. Error was coded “1” when MRI and CT disagreed, and coded “0” otherwise. This analysis was performed *per*-reader and *per*-sequence. Then, we assessed the performance of pseudo-CT MRI sequences in detecting osteolytic lesions, taking the consensus CT reading as a reference. True positives (TP), true negatives (TN), false positives (FP), and false negative (FN) were reported to calculate sensitivity (= TP/(TP + FN)*100), specificity (= TN/(TN + FP)*100), positive predictive value (TP/(TP + FP)*100), negative predictive value (= TN/(TN + FN)*100), and the overall accuracy (= (TP + TN)/(TP + FP + TN + FN)*100). This analysis was performed *per*-reader, *per*-sequence and *per*-region.

The histograms of differences in quantitative scoring were computed as follows: “ZTE-DLCSC score minus ZTE score,” “ZTE-DLCSC score minus BB score,” “ZTE-DLCSC score minus CT score,” “ZTE score minus CT score,” and “BB score minus CT score.” Then, percentages of positive and negative differences (indicating a larger number of lesions detected by one or the other sequence) were reported. This analysis was performed *per*-region using R1’s measurements. A Wilcoxon paired test was used to assess the difference between sequences in terms of percentages of positive and negative differences (considering all regions).

The effect of the three factors, sequence, region and reader, on the accuracy was assessed using a 3-way mixed analysis of variance‒ANOVA model, where fixed effects were associated with the sequence and region factors, and random effects were associated with the reader factor. An interaction term “sequence*reader” was also included. Then, a Tukey comparison procedure was implemented to perform pairwise comparisons of Acc between sequences (*per*-reader, considering all regions).

Statistical significance was declared at *p* < 0.05 for all tests. All calculations were done using StatsDirect v4.0.4 software (StatsDirect Ltd.) and MATLAB R2024b (MathWorks).

### Adjudication of readings

As a last step of image review, the three readers determined in consensus the causes of FP and FN of osteolytic MM lesions from pseudo-CT images, as identified during the data and statistical analysis. This was done using all available MRI sequences (including pseudo-CT sequences, but also 3D fast spin-echo T1, 3D T1 Dixon, diffusion-weighted sequences and apparent diffusion coefficient maps not used in previous readings) and the reference CT.

## Results

### Patient characteristics

**Fig. 1:**
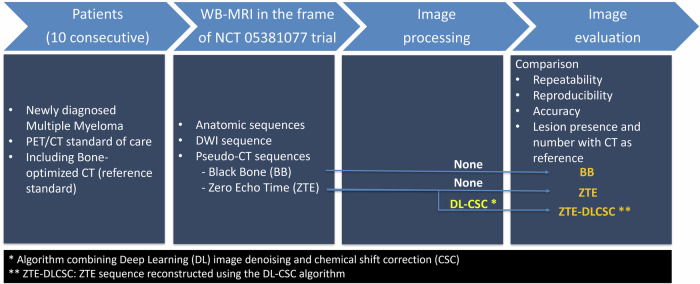
Study flowchart illustrating the study design, including the patient population, the inclusion criteria, the image acquisition process, and the image processing and reading pipeline

Ten patients (7 males, 3 females), aged 67 ± 12 years (mean ± standard deviation), underwent a whole-body MRI examination including pseudo-CT MRI sequences (ZTE and BB). All had undergone a ^18^F-FDG-PET/CT within 2 weeks of the MRI examination (Fig. 1). The distribution of lytic lesions observed at CT in the ten skeletal areas was as follows: % of positive (total number of lesions): L1, 50% (*n* = 13); L2, 60% (*n* = 13); L3, 30% (*n* = 11); L4, 50% (*n* = 9); L5, 40% (*n* = 9); sacrum, 60% (*n* = 17); right ilium, 90% (*n* = 30); left ilium, 80% (*n* = 30); right femur, 50% (*n* = 10); left femur, 50% (*n* = 12).

### Repeatability for lesion detection (Table 2)

Using ZTE, repeatability ranged from moderate (AC1 = 0.45 in L4) to very good (AC1 = 1.00 in the right femur); repeatability was good in eight out of ten regions. Using ZTE-DLCSC, repeatability ranged from good (AC1 ≥ 0.60 in five regions) to very good (AC1 = 1.00 in the other five regions). Using BB, repeatability ranged from good (AC1 ≥ 0.60 in five regions) to very good (0.80 ≤ AC1 ≤ 0.82 in the other five regions). Using CT, repeatability was at least very good regardless of the anatomical region (all AC1 ≥ 0.80).

**Table 2:** Repeatability of the categorical score (presence/absence of osteolytic lesion) from reader R2

Region	ZTE	ZTE-DLCSC	BB	CT
L1	0.60 [0.10; 1.10]	0.60 [0.10; 1.10]	0.80 [0.43; 1.17]	1.00 [1.00; 1.00]
L2	0.65 [0.18; 1.13]	0.66 [0.18; 1.13]	0.80 [0.43; 1.17]	1.00 [1.00; 1.00]
L3	0.80 [0.43; 1.17]	1.00 [1.00; 1.00]	0.60 [0.10; 1.10]	1.00 [1.00; 1.00]
L4	0.45 [-0.13; 1.03]	1.00 [1.00; 1.00]	0.62 [0.12; 1.11]	0.80 [0.43; 1.17]
L5	0.60 [0.10; 1.10]	0.66 [0.18; 1.13]	0.71 [0.28; 1.13]	1.00 [1.00; 1.00]
Sacrum	0.66 [0.18; 1.13]	1.00 [1.00; 1.00]	0.66 [0.18; 1.13]	0.80 [0.43; 1.17]
Right ilium	0.80 [0.43; 1.17]	1.00 [1.00; 1.00]	0.82 [0.46; 1.17]	0.87 [0.59; 1.14]
Left ilium	0.62 [0.12; 1.11]	0.71 [0.28; 1.13]	0.71 [0.28; 1.13]	0.87 [0.59; 1.14]
Right femur	1.00 [1.00; 1.00]	1.00 [1.00; 1.00]	0.82 [0.46; 1.17]	0.80 [0.43; 1.17]
Left femur	0.71 [0.28; 1.13]	0.80 [0.43; 1.18]	0.82 [0.46; 1.17]	0.80 [0.43; 1.17]

The table reads as follows: in the lumbar vertebra L1 (1st line), repeatability was good using ZTE (AC1 = 0.60) and ZTE-DLCSC (AC1 = 0.60) sequences, while it was very good using the BB sequence (AC1 = 0.80). Using CT for assessing L1, reader R2 achieved a perfect intra-reader agreement (AC1 = 1.00); 95% CIs are given in brackets

*BB* Black bone sequence, *ZTE* Zero echo time sequence, *ZTE-DLCSC* ZTE sequence reconstructed with deep learning and chemical shift correction algorithm

### Reproducibility for lesion detection (Table 3)

Using ZTE, reproducibility was fair to moderate in the spine and sacrum (0.21 ≤ AC1 ≤ 0.54), and good to very good in the ilium and femurs (0.61 ≤ AC1 ≤ 1.00). Using ZTE-DLCSC, reproducibility ranged from good to very good in all regions (0.60 ≤ AC1 ≤ 1.00), though it was only moderate in the right ilium and left femur (AC1 = 0.56 and 0.47, respectively). Using BB, reproducibility ranged from fair to moderate in all regions (0.20 ≤ AC1 ≤ 0.59), though it was good in the left femur (AC1 = 0.74). Using CT, reproducibility was at least very good in all regions (all AC1 ≥ 0.80).

**Table 3:** Reproducibility of the categorical score (presence/absence of osteolytic lesion) assessed from the 1st reading from all readers

Region	AC2			
	ZTE	ZTE-DLCSC	BB	CT
L1	0.47 [-0.04; 0.98]	0.60 [0.14; 1.00]	0.20 [-0.29; 0.69]	0.87 [0.57; 1.00]
L2	0.54 [0.02; 1.00]	0.76 [0.35; 1.00]	0.47 [-0.04; 0.98]	1.00 [1.00; 1.00]
L3	0.35 [-0.17; 0.86]	0.60 [0.14; 1.00]	0.20 [-0.29; 0.69]	0.88 [0.60; 1.00]
L4	0.25 [-0.29; 0.79]	0.87 [0.57; 1.00]	0.36 [-0.14; 0.86]	0.87 [0.57; 1.00]
L5	0.21 [-0.26; 0.69]	1.00 [1.00; 1.00]	0.56 [0.07; 1.00]	1.00 [1.00; 1.00]
Sacrum	0.50 [0.01; 1.00]	0.87 [0.57; 1.00]	0.52 [-0.03; 1.00]	0.87 [0.57; 1.00]
Right ilium	0.87 [0.57; 1.00]	0.56 [0.07; 1.00]	0.40 [-0.15; 0.95]	0.91 [0.71; 1.00]
Left ilium	0.61 [0.15; 1.00]	0.90 [0.65; 1.00]	0.59 [0.05; 1.00]	0.80 [0.46; 1.00]
Right femur	1.00 [1.00; 1.00]	0.62 [0.17; 1.00]	0.47 [-0.02; 0.96]	0.87 [0.57; 1.00]
Left femur	0.67 [0.21; 1.00]	0.47 [-0.02; 0.96]	0.74 [0.33; 1.00]	0.87 [0.57; 1.00]

The table reads as follows: in the lumbar vertebra L1 (1st line), reproducibility ranged from fair (BB), moderate (ZTE), good (ZTE-DLCSC) to very good (CT). All regions considered, the reproducibility was at worst fair using ZTE or BB, good using ZTE-DLCSC (except in the right ilium and left femur without a clear explanation) and very good using CT; 95% confidence intervals are given in brackets

*BB* Black bone sequence, *ZTE* Zero echo time sequence, *ZTE-DLCSC* ZTE sequence reconstructed with deep learning and chemical shift correction algorithm

### Accuracy of pseudo-CT sequences for lesion detection (Table 4a–c)

Goodman-Kruskal test [32] showed that there is little evidence of an association between regions and patients in terms of error in using MRI compared to CT for lesion detection (Supplementary Table S1). Bone regions were thus treated as independent observations.

**Table 4:** Diagnostic accuracy of the ZTE sequence (**a**), the ZTE-DLCSC sequence (**b**), and the BB sequence (**c**) in detecting osteolytic lesions

a. Diagnostic accuracy of the ZTE sequence in detecting osteolytic lesions					
	Reader 1				
Region	Sensitivity	Specificity	PPV	NPV	Accuracy
	TP	TN	FP	FN	
L1	40 [5.3; 85]	80 [28; 99]	67 [9.4; 99]	57 [18; 90]	60 [26; 88]
	2	4	1	3	
L2	83 [36; 100]	25 [0.6; 81]	63 [24; 91]	50 [1.3; 99]	60 [26; 88]
	5	1	3	1	
L3	67 [9.4; 99]	71 [29; 96]	50 [6.8; 93]	83 [36; 100]	70 [35; 93]
	2	5	2	1	
L4	20 [1.0; 72]	80 [28; 99]	50 [1.3; 99]	50 [16; 84]	50 [19; 81]
	1	4	1	4	
L5	25 [1.0; 81]	67 [22; 96]	33 [1.0; 91]	57 [18; 90]	50 [19; 81]
	1	4	2	3	
Sacrum	83 [36; 100]	50 [6.8; 93]	71 [29; 96]	67 [9.4; 99]	70 [35; 93]
	5	2	2	1	
Right ilium	56 [21; 86]	0.0 [0.0; 98]	83 [36; 100]	0.0 [0.0; 60]	50 [19; 81]
	5	0	1	4	
Left ilium	75 [35; 97]	100 [16; 100]	100 [54; 100]	50 [6.8; 93]	80 [44; 97]
	6	2	0	2	
Right femur	20 [1.0; 72]	80 [28; 99]	50 [1.3; 99]	50 [16; 84]	50 [19; 81]
	1	4	1	4	
Left femur	60 [15; 95]	80 [28; 99]	75 [19; 99]	67 [22; 96]	70 [35; 93]
	3	4	1	2	

	Reader 2				
Region	Sensitivity	Specificity	PPV	NPV	Accuracy
	TP	TN	FP	FN	
L1	40 [5.3; 85]	40 [5.3; 85]	40 [5.3; 85]	40 [5.3; 85]	40 [12; 74]
	2	2	3	3	
L2	83 [36; 100]	75 [19; 99]	83 [36; 100]	75 [19; 99]	80 [44; 97]
	5	3	1	1	
L3	67 [9.4; 99]	71 [29; 96]	50 [6.8; 96]	83 [36; 100]	70 [35; 93]
	2	5	2	1	
L4	40 [5.3; 85]	40 [5.3; 85]	40 [5.3; 85]	40 [5.3; 85]	40 [12; 74]
	2	2	3	3	
L5	50 [6.8; 93]	33 [4.3; 78]	33 [4.3; 78]	50 [6.8; 93]	40 [12; 74]
	2	2	4	2	
Sacrum	100 [54; 100]	75 [19; 99]	86 [42; 100]	100 [29; 100]	90 [55; 100]
	6	3	1	0	
Right ilium	44 [19; 73]	0.0 [0.0; 98]	80 [28; 99]	0.0 [0.0; 52]	40 [12; 74]
	4	0	1	5	
Left ilium	75 [35; 97]	100 [16; 100]	100 [54; 100]	50 [6.8; 93]	80 [44; 97]
	6	2	0	2	
Right femur	20 [1.0; 72]	80 [28; 99]	50 [1.3; 99]	50 [16; 84]	50 [19; 81]
	1	4	1	4	
Left femur	0.0 [0.0; 52]	80 [28; 99]	0.0 [0.0; 98]	44 [14; 79]	40 [12; 74]
	0	4	1	5	

	Reader 3				
Region	Sensitivity	Specificity	PPV	NPV	Accuracy
	TP	TN	FP	FN	
L1	60 [15; 95]	40 [5.3; 85]	50 [12; 88]	50 [6.8; 93]	50 [19; 81]
	3	2	3	2	
L2	83 [36; 96]	50 [6.8; 93]	71 [29; 96]	67 [9.4; 99]	70 [35; 93]
	5	2	2	1	
L3	100 [29; 100]	71 [29; 96]	60 [15; 95]	100 [48; 100]	60 [26; 88]
	3	5	2	0	
L4	80 [28; 99]	100 [48; 100]	100 [40; 100]	83 [36; 100]	60 [26; 88]
	4	5	0	1	
L5	75 [19; 99]	83 [36; 100]	75 [19; 99]	83 [36; 100]	80 [44; 97]
	3	5	1	1	
Sacrum	50 [12; 88]	50 [6.8; 93]	60 [15; 95]	40 [5.3; 85]	50 [19; 81]
	3	2	2	3	
Right ilium	56 [21; 86]	0.0 [0.0; 98]	83 [36; 100]	0.0 [0.0; 60]	50 [19; 81]
	5	0	1	4	
Left ilium	63 [24; 91]	100 [16; 100]	100 [48; 100]	40 [53; 85]	70 [35; 93]
	5	2	0	3	
Right femur	20 [10; 72]	80 [28; 99]	50 [1.3; 99]	50 [16; 84]	50 [19; 81]
	1	4	1	4	
Left femur	40 [5.3; 85]	80 [28; 99]	67 [9.4; 99]	57 [18; 90]	60 [26; 88]
	2	4	1	3	

b. Diagnostic accuracy of the ZTE-DLCSC sequence in detecting osteolytic lesions					
	Reader 1				
Region	Sensitivity	Specificity	PPV	NPV	Accuracy
	TP	TN	FP	FN	
L1	80 [28; 99]	100 [15; 95]	80 [28; 99]	80 [28; 99]	80 [44; 97]
	4	4	1	1	
L2	100 [54; 100]	100 [40; 100]	100 [54; 100]	100 [40; 100]	100 [69; 100]
	6	4	0	0	
L3	100 [29; 100]	71 [29; 96]	60 [15; 95]	100 [15; 95]	80 [44; 97]
	3	5	2	0	
L4	100 [15; 95]	80 [28; 99]	83 [36; 100]	100 [40; 100]	90 [55; 100]
	5	4	1	0	
L5	100 [40; 100]	80 [54; 100]	100 [40; 100]	80 [54; 100]	100 [69; 100]
	4	6	0	0	
Sacrum	83 [36; 100]	80 [28; 99]	100 [48; 100]	80 [28; 99]	90 [55; 100]
	5	4	0	1	
Right ilium	100 [66; 100]	100 [2.5; 100]	100 [66; 100]	100 [2.5; 100]	100 [69; 100]
	9	1	0	0	
Left ilium	100 [63; 100]	100 [16; 100]	100 [63; 100]	100 [16; 100]	100 [69; 100]
	8	2	0	0	
Right femur	100 [54; 100]	100 [40; 100]	100 [54; 100]	100 [40; 100]	100 [69; 100]
	6	4	0	0	
Left femur	100 [48; 100]	100 [48; 100]	100 [48; 100]	100 [48; 100]	100 [69; 100]
	5	5	0	0	

	Reader 2				
Region	Sensitivity	Specificity	PPV	NPV	Accuracy
	TP	TN	FP	FN	
L1	60 [15; 95]	60 [15; 95]	60 [15; 95]	60 [15; 95]	60 [26; 88]
	3	3	2	2	
L2	100 [54; 100]	50 [6.8; 93]	75 [35; 97]	100 [16; 100]	80 [44; 97]
	6	2	2	0	
L3	100 [29; 100]	71 [29; 96]	60 [15; 95]	100 [48; 100]	80 [44; 97]
	3	5	2	0	
L4	100 [48; 100]	100 [48; 100]	100 [48; 100]	100 [48; 100]	100 [69; 100]
	5	5	0	0	
L5	100 [40; 100]	100 [54; 100]	100 [40; 100]	100 [54; 100]	100 [69; 100]
	4	6	0	0	
Sacrum	83 [36; 100]	100 [40; 100]	100 [48; 100]	80 [28; 99]	90 [55; 100]
	5	4	0	1	
Right ilium	56 [21; 86]	100 [2.5; 100]	100 [48; 100]	25 [0.6; 81]	60 [26; 88]
	5	1	0	4	
Left ilium	100 [63; 100]	100 [16; 100]	100 [63; 100]	100 [16; 100]	100 [69; 100]
	8	2	0	0	
Right femur	100 [48; 100]	40 [5.3; 85]	63 [24; 91]	100 [16; 100]	70 [35; 100]
	5	2	3	0	
Left femur	60 [15; 95]	60 [15; 95]	60 [15; 95]	60 [15; 95]	60 [26; 88]
	3	3	2	2	

	Reader 3				
Region	Sensitivity	Specificity	PPV	NPV	Accuracy
	TP	TN	FP	FN	
L1	60 [15; 95]	60 [15; 95]	50 [12; 88]	50 [6.8; 93]	50 [19; 81]
	3	2	3	2	
L2	100 [54; 100]	100 [40; 100]	100 [54; 100]	100 [40; 100]	100 [69; 100]
	6	4	0	0	
L3	100 [29; 100]	86 [42; 100]	75 [19; 99]	100 [54; 100]	90 [55; 100]
	3	6	1	0	
L4	100 [48; 100]	80 [28; 99]	83 [36; 100]	100 [40; 100]	90 [55; 100]
	5	4	1	0	
L5	100 [40; 100]	100 [54; 100]	100 [40; 100]	100 [54; 100]	100 [69; 100]
	4	6	0	0	
Sacrum	67 [22; 96]	100 [40; 100]	100 [40; 100]	67 [22; 96]	80 [44; 97]
	4	4	0	2	
Right ilium	89 [52; 100]	100 [2.5; 100]	100 [63; 100]	50 [1.3; 99]	90 [55; 100]
	8	1	0	1	
Left ilium	88 [47; 100]	100 [16; 100]	100 [59; 100]	67 [9.4; 99]	90 [55; 100]
	7	2	0	1	
Right femur	80 [28; 99]	60 [15; 95]	67 [22; 96]	75 [19; 99]	70 [35; 93]
	4	3	2	1	
Left femur	100 [48; 100]	80 [28; 99]	83 [36; 100]	100 [40; 100]	90 [55; 100]
	5	4	1	0	

c. Diagnostic accuracy of the BB sequence in detecting osteolytic lesions					
	Reader 1				
Region	Sensitivity	Specificity	PPV	NPV	Accuracy
	TP	TN	FP	FN	
L1	80 [28; 99]	100 [48; 100]	100 [40; 100]	83 [36; 100]	90 [55; 100]
	4	5	0	1	
L2	67 [22; 96]	75 [19; 99]	80 [28; 99]	60 [15; 95]	70 [35; 93]
	4	3	1	2	
L3	100 [29; 100]	71 [29; 96]	60 [15; 95]	100 [48; 100]	80 [44; 97]
	3	5	2	0	
L4	40 [5.3; 85]	100 [48; 100]	100 [16; 100]	63 [24; 91]	70 [35; 93]
	2	5	0	3	
L5	25 [1.0; 81]	100 [54; 100]	100 [2.5; 100]	67 [30; 93]	70 [35; 93]
	1	6	0	3	
Sacrum	83 [36; 100]	50 [6.8; 93]	71 [29; 96]	67 [9.4; 99]	70 [35; 93]
	5	2	2	1	
Right ilium	67 [30; 93]	100 [2.5; 100]	100 [54; 100]	75 [19; 99]	70 [35; 93]
	6	1	0	3	
Left ilium	75 [35; 97]	50 [1.3; 99]	86 [42; 100]	70 [35; 93]	70 [35; 93]
	6	1	1	2	
Right femur	80 [28; 99]	60 [15; 95]	67 [22; 96]	75 [19; 99]	70 [35; 93]
	4	3	2	1	
Left femur	80 [28; 99]	80 [28; 99]	80 [28; 99]	80 [28; 99]	80 [44; 97]
	4	4	1	1	

	Reader 2				
Region	Sensitivity	Specificity	PPV	NPV	Accuracy
	TP	TN	FP	FN	
L1	60 [15; 95]	60 [15; 95]	60 [15; 95]	60 [15; 95]	60 [26; 88]
	3	3	2	2	
L2	67 [22; 96]	75 [19; 99]	80 [28; 99]	60 [15; 95]	70 [35; 93]
	4	3	1	2	
L3	67 [9.4; 99]	71 [29; 96]	50 [6.8; 93]	83 [36; 100]	70 [35; 93]
	2	5	2	1	
L4	60 [15; 95]	80 [28; 99]	75 [19; 99]	67 [22; 96]	70 [35; 93]
	3	4	1	2	
L5	50 [6.8; 93]	83 [36; 100]	67 [9.4; 99]	71 [29; 96]	70 [35; 93]
	2	5	1	2	
Sacrum	67 [22; 96]	50 [6.8; 93]	67 [22; 96]	50 [6.8; 93]	60 [26; 88]
	4	2	2	2	
Right ilium	78 [40; 97]	100 [2.5; 100]	100 [59; 100]	33 [0.8; 91]	80 [44; 97]
	7	1	0	2	
Left ilium	88 [47; 100]	50 [1.3; 99]	88 [47; 100]	50 [1.3; 99]	80 [44; 97]
	7	1	1	1	
Right femur	40 [5.3; 85]	80 [28; 99]	67 [9.4; 99]	57 [18; 90]	60 [26; 88]
	2	4	1	3	
Left femur	40 [5.3; 85]	80 [28; 99]	67 [9.4; 99]	57 [18; 90]	60 [26; 88]
	2	4	1	3	

	Reader 3				
Region	Sensitivity	Specificity	PPV	NPV	Accuracy
	TP	TN	FP	FN	
L1	80 [28; 99]	40 [5.3; 85]	57 [18; 90]	67 [9.4; 99]	60 [26; 88]
	4	2	3	1	
L2	50 [12; 88]	75 [19; 99]	75 [19; 99]	50 [12; 88]	60 [26; 88]
	3	3	1	3	
L3	100 [29; 100]	71 [29; 96]	60 [15; 95]	100 [48; 100]	80 [44; 97]
	3	5	2	0	
L4	100 [48; 100]	80 [28; 99]	83 [36; 96]	100 [40; 100]	90 [55; 100]
	5	4	1	0	
L5	100 [40; 100]	100 [54; 100]	100 [40; 100]	100 [54; 100]	100 [69; 100]
	4	6	0	0	
Sacrum	83 [36; 100]	50 [6.8; 93]	71 [29; 96]	67 [9.4; 99]	70 [35; 93]
	5	2	2	1	
Right ilium	67 [30; 93]	0.0 [0.0; 98]	86 [42; 100]	0.0 [0.0; 71]	60 [26; 88]
	6	0	1	3	
Left ilium	100 [63; 100]	100 [16; 100]	100 [63; 100]	100 [16; 100]	100 [69; 100]
	8	2	0	0	
Right femur	60 [15; 95]	60 [15; 95]	60 [15; 95]	60 [15; 95]	60 [26; 88]
	3	3	2	2	
Left femur	80 [28; 99]	80 [28; 99]	80 [28; 99]	80 [28; 99]	80 [44; 97]
	4	4	1	1	

Performance is assessed from the 1st reading from all readers. True positives (TP), false positives (FP), false negatives (FN), true negatives (TN), sensitivity, specificity, positive predictive value (PPV), negative predictive value (NPV) and accuracy are reported; 95% confidence intervals are given in brackets

*BB* Black bone sequence, *ZTE* Zero echo time sequence, *ZTE-DLCSC* ZTE sequence reconstructed with deep learning and chemical shift correction algorithm

Using ZTE, depending on the region, the accuracy ranged 50‒80% (mean accuracy for all regions = 61%) for R1, 40‒90% (mean accuracy for all regions = 57%) for R2, and 50‒80% (mean accuracy for all regions = 60%) for R3. Using ZTE-DLCSC, the same data were 80‒100% (93%) for R1, 60‒100% (80%) for R2, and 50‒100% (85%) for R3, respectively. Using BB, the same data were 70‒90% (74%) for R1, 60‒80% (66%) for R2, and 60‒100% (76%) for R3, respectively.

### Comparison of accuracy between pseudo-CT sequences

The three-way mixed analysis of variance showed that the sequence and region factors have an effect on the accuracy (*p*^Sequence^ = 0.001, *p*^Region^ = 0.001). There is little evidence that the reader factor has an effect (*p*^Reader^ = 0.070). Likewise, there is little evidence that the effect of the sequence depends on the reader (*p*^Sequence*Reader^ = 0.573). In senior reader R1 (considering all regions), the accuracy of ZTE-DLCSC was higher than that of ZTE (+32%, *p* < 0.001), the accuracy of ZTE-DLCSC was higher than that of BB (+19%, *p* < 0.001), and the accuracy of BB was higher than that of ZTE (+13%, *p* = 0.008).

**Fig. 2:**
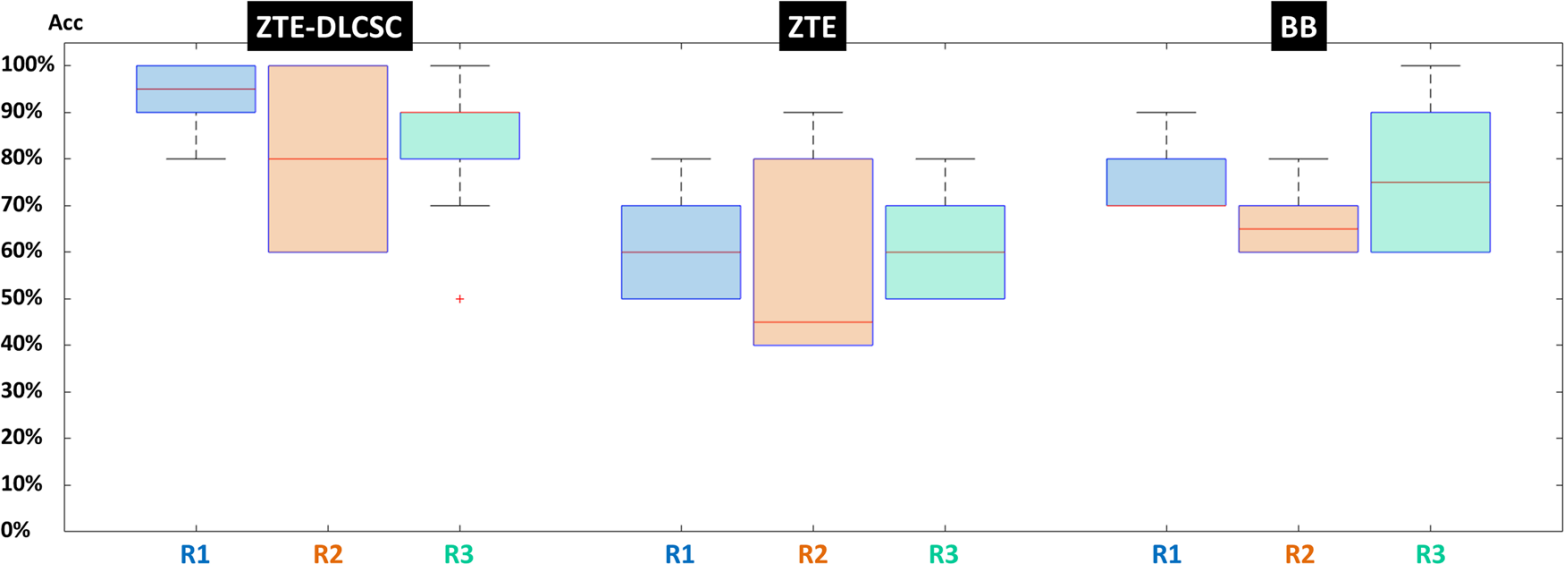
Boxplot of accuracy (Acc) of pseudo-CT MRI sequences in detecting osteolytic lesions according to each reader, taking CT as reference (see Table 4a–c). BB, Black bone pseudo-CT sequence; CT, Computed tomography; ZTE, Zero echo time pseudo-CT sequence; ZTE-DLCSC, ZTE sequence reconstructed with deep learning and chemical shift correction algorithm

For junior reader R2, the accuracy of ZTE-DLCSC was higher than that of ZTE (+23%, *p* = 0.010), the accuracy of ZTE-DLCSC was not significantly different from that of BB (*p* = 0.140), and the accuracy of BB was not significantly different from that of ZTE (*p* = 0.428). For junior reader R3, the accuracy of ZTE-DLCSC was higher than that of ZTE (+25%, *p* = 0.002), the accuracy of ZTE-DLCSC was not significantly different from that of BB (*p* = 0.349), and the accuracy of BB was higher than that of ZTE (+16%, *p* = 0.047) (Fig. 2).

### Adjudication of FP and FN readings for osteolytic lesions (Figs. 3–5)

During the consensus session, FN and FP readings for lytic MM foci were analyzed in detail. ZTE showed the highest FN rate (103 regions), mostly due to technical limitations (60%) and missed lesions (27%), whereas ZTE-DLCSC reduced FN findings to 32 regions, mainly related to reader oversight (59%). BB yielded 70 FN regions, with nearly half caused by missed lesions (47%). For FP findings, ZTE produced 63 regions, primarily due to pseudo-lytic artifacts (46%) and benign lesions misclassified as malignant (41%). ZTE-DLCSC showed 33 FP regions, mostly benign overcalls (61%), and BB presented 51 FP regions, dominated by benign misclassifications (71%). Detailed distributions of the causes of FN and FP findings across readers and sequences were determined during the consensus session and are reported in Supplementary Table S2.

**Fig. 3:**
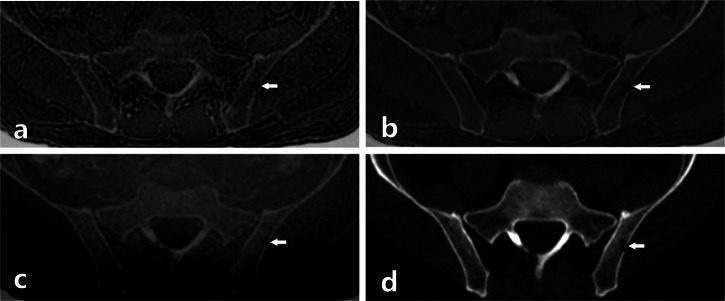
Images of the pelvis of a 65-year-old patient with newly diagnosed MM. Axial ZTE (**a**), ZTE-DLCSC (**b**), BB (**c**), and CT (**d**) reconstructions. In the left ilium, a small osteolytic lesion (arrows) was only detected by readers on CT (**d**) and ZTE-DLCSC (**b**). Retrospectively, the lesion is also visible on ZTE (**a**) and BB (**c**), though it is more difficult to distinguish due to the low signal-to-noise of the image and chemical shift artifacts. BB, Black bone pseudo-CT sequence; CT, Computed tomography; MM, Multiple myeloma; ZTE, Zero echo time pseudo-CT sequence; ZTE-DLCSC, ZTE sequence reconstructed with deep learning and chemical shift correction algorithm

**Fig. 4:**
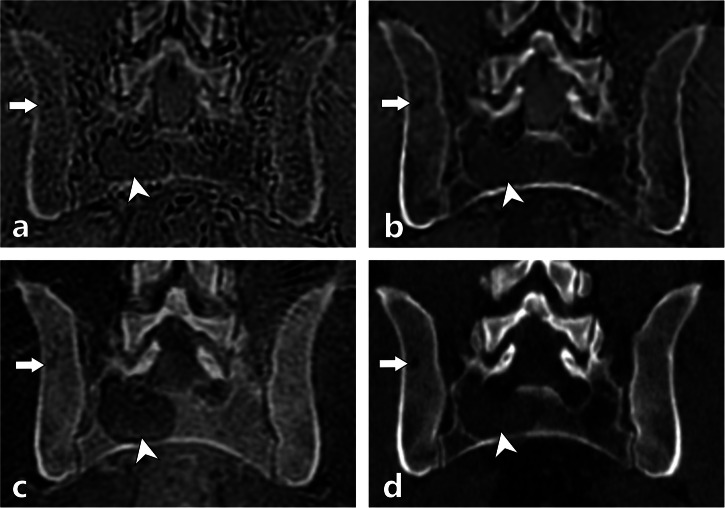
Images of the pelvis in a 49-year-old patient with newly diagnosed MM. Coronal reconstructed ZTE (**a**), ZTE-DLCSC (**b**), BB (**c**), and CT (**d**) images. In the right part of the sacrum, a large osteolytic lesion is clearly visible on all sequences (arrowheads). In the right ilium, a smaller osteolytic lesion (arrows) was detected on all sequences except ZTE (**a**), where it was missed by all readers due to technical limitations of the ZTE sequence (false negative due to “undetectable” lesion). BB, Black bone pseudo-CT sequence; CT, Computed tomography; MM, Multiple myeloma; ZTE, Zero echo time pseudo-CT sequence; ZTE-DLCSC, ZTE sequence reconstructed with deep learning and chemical shift correction algorithm

**Fig. 5:**
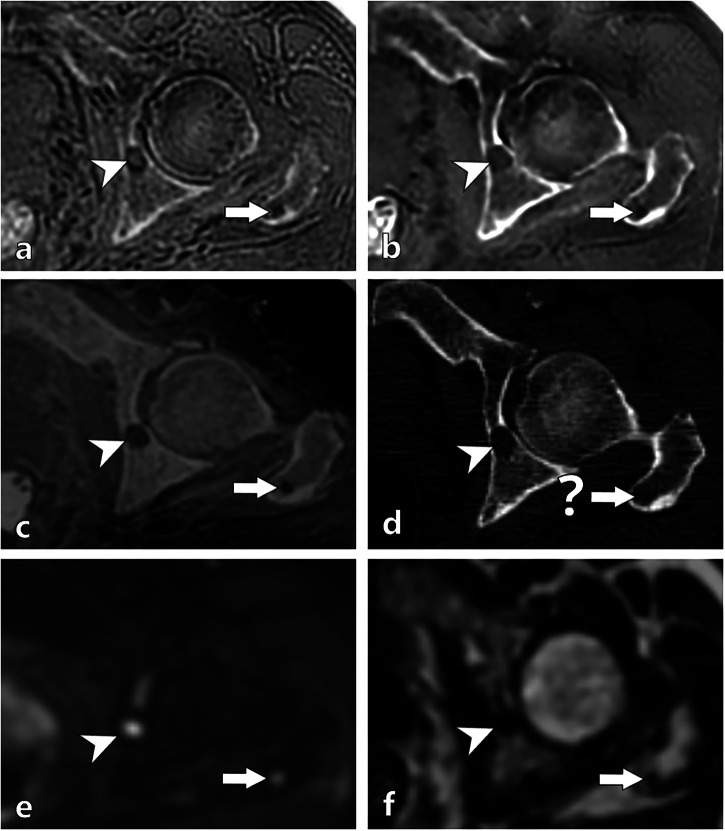
Images of the left ilium and femur in a 63-year-old with newly diagnosed MM. Axial reconstructed images of ZTE (**a**), ZTE-DLCSC (**b**), BB (**c**), CT (**d**), DWI (**e**) and Dixon T1 fat (**f**). All pseudo-CT MRI sequences (**a**–**c**) reveal a lytic lesion within the left ilium (arrowhead), which is also visible on CT (**d**). In the greater trochanter of the left femur (arrow), all pseudo-CT MRI sequences (**a**–**c**) show a lytic focus that was undetectable on CT (**d**). The standard MRI sequences DWI (**e**) and Dixon T1 fat (**f**) confirm the existence of the iliac lesion (arrowhead), but also of the femoral lesion (arrow) (suggesting a false negative of the reference CT). BB, Black bone pseudo-CT sequence; CT, Computed tomography; MM, Multiple myeloma; ZTE, Zero echo time pseudo-CT sequence; ZTE-DLCSC, ZTE sequence reconstructed with deep learning and chemical shift correction algorithm

### Quantitative scoring (histograms of differences in lesion count between ZTE-DLCSC and other sequences)

**Fig. 6:**
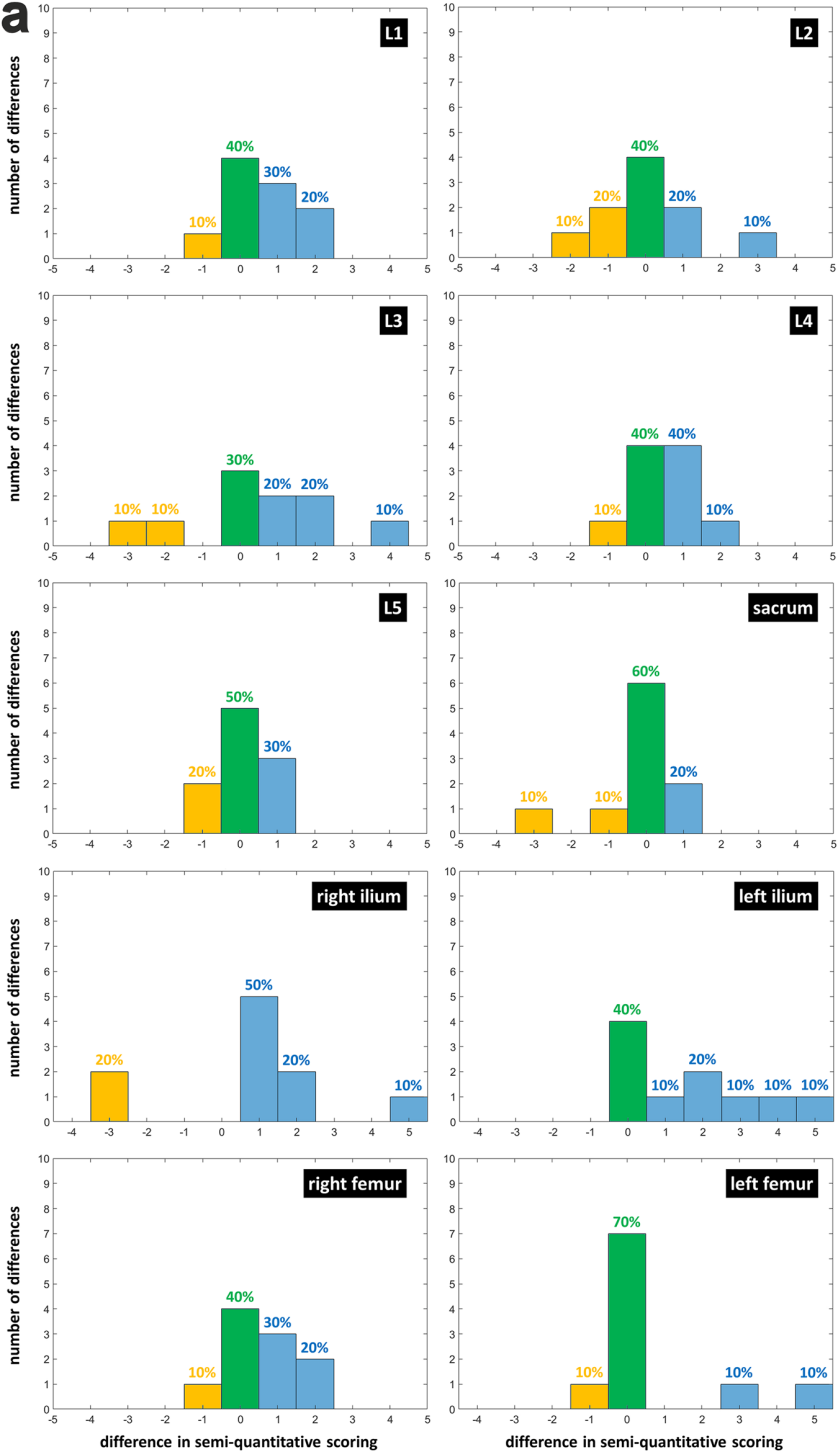

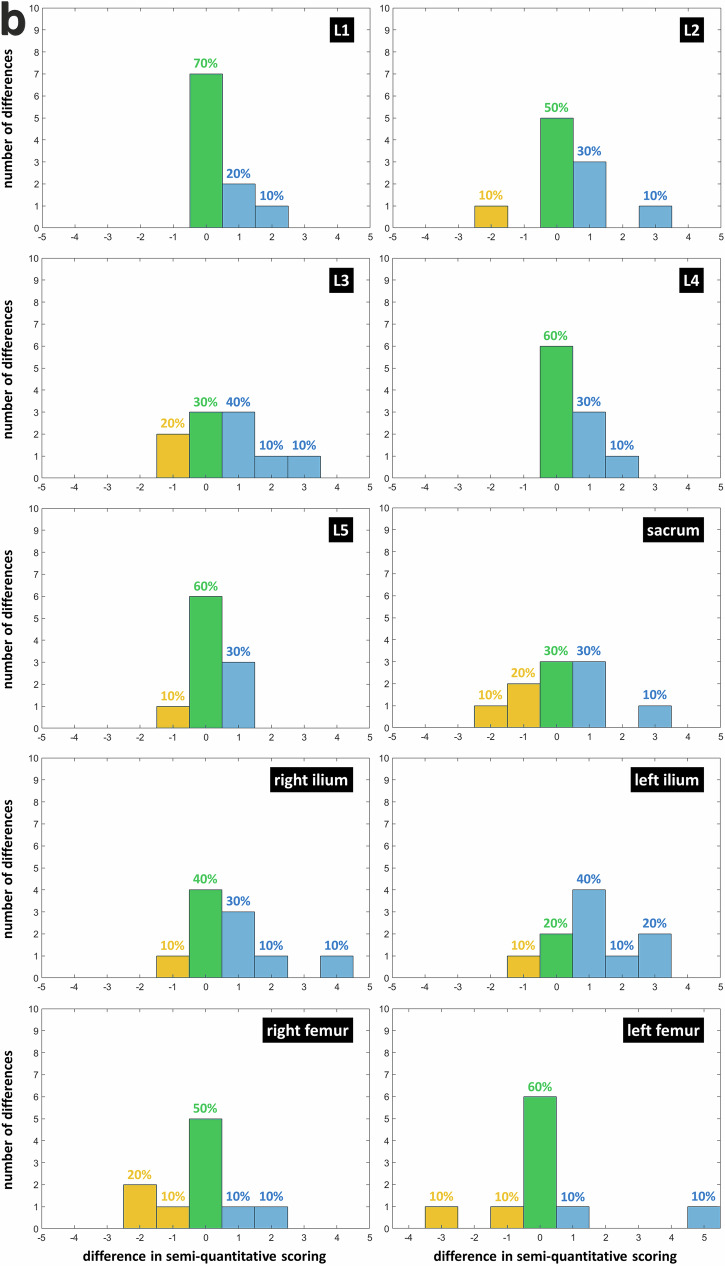

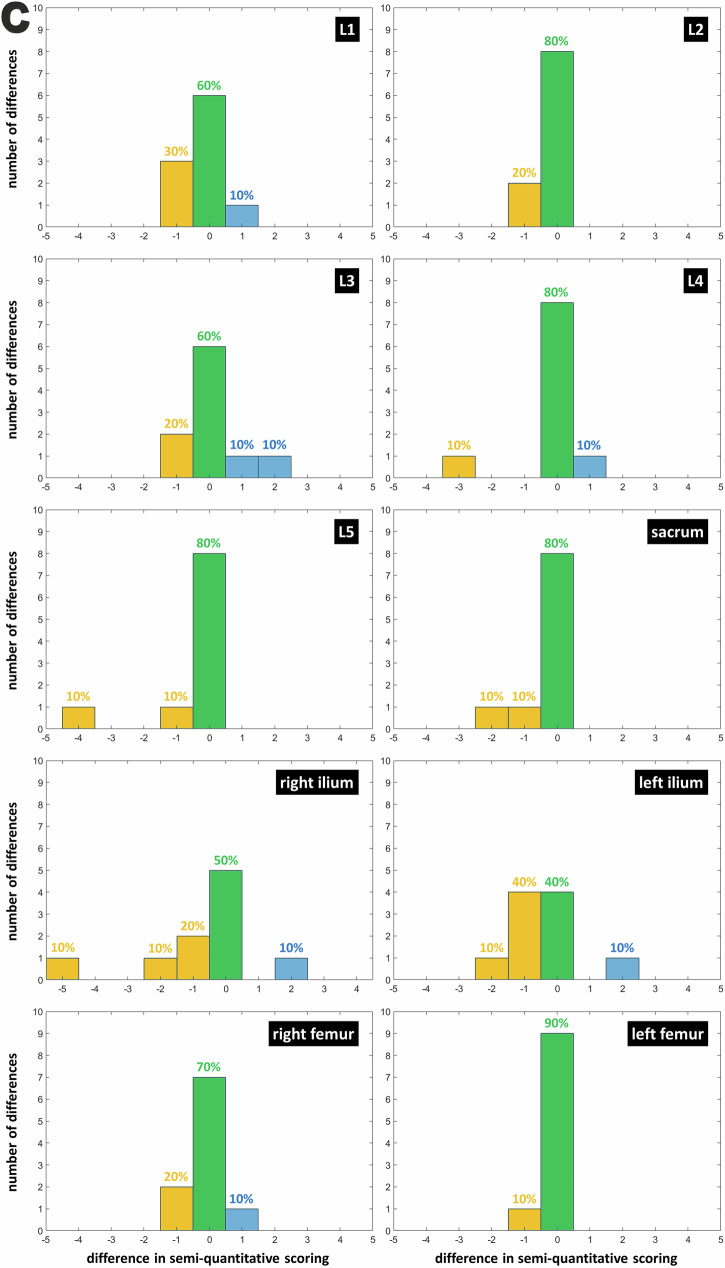
**a** Histograms of the differences computed as “ZTE-DLCSC score minus ZTE score” for the quantitative score of osteolytic lesions. Measurements from senior reader R1 are used. Blue bars represent positive differences corresponding to a higher number of lesions detected by ZTE-DLCSC. Null differences, which correspond to the same number of lesions detected by both sequences, are represented by green bars. Negative differences, which correspond to a lower number of lesions detected by ZTE-DLCSC, are represented by yellow bars. BB, Black bone pseudo-CT sequence; CT, Computed tomography; MM, Multiple myeloma; ZTE, Zero echo time pseudo-CT sequence; ZTE-DLCSC, ZTE sequence reconstructed with deep learning and chemical shift correction algorithm. **b** Histograms of the differences computed as “ZTE-DLCSC score minus BB score” in the quantitative score of osteolytic lesions. **c** Histograms of the differences computed as “ZTE-DLCSC score minus CT score” for the quantitative score of osteolytic lesions

The ZTE-DLCSC *versus* ZTE comparison shows that differences in quantitative score were either positive (50%) or null (40%). This indicates that, when a different number of lesions were detected, cases in which the ZTE-DLCSC detected more lesions were more frequent (difference in median frequency = +30%, *p* = 0.011, Wilcoxon) (Fig. 6a). The same trend was observed in the ZTE-DLCSC *versus* BB comparison, with null or positive differences (50% and 40%, respectively), indicating that, when a different number of lesions were detected, cases in which the ZTE-DLCSC detected more lesions were more frequent (difference in median frequency = +25%, *p* = 0.024, Wilcoxon) (Fig. 6b). The ZTE-DLCSC *versus* CT comparison shows that differences were mostly null (75%), with cases in which CT detects more lesions being slightly more frequent (difference in median frequency = +15%, *p* = 0.011, Wilcoxon) (Fig. 6c).

## Discussion

This study demonstrated that the use of the DL-CSC reconstruction algorithm significantly improves the reproducibility and repeatability of the ZTE sequence for detecting osteolytic lesions in MM patients. It also showed that the DL-CSC algorithm improves the diagnostic accuracy of the ZTE sequence. The ZTE-DLCSC sequences showed significantly higher accuracy compared to native ZTE in all readers and compared to BB in the senior reader. The accuracy of BB was higher than that of native ZTE for two readers, which aligns with a preliminary study showing slightly higher accuracy for BB than the standard ZTE [16].

The DL-CSC algorithm addresses the limitations of the ZTE sequence, such as limited signal and image quality (especially in the spine) and CS artifacts, which were identified in a preliminary study [16]. The better image quality and lesion definition achieved with DL decreased the number of FP (*e.g*., considering a benign lesion malignant) and FN (missing a lesion due to a lack of signal). The CSC, by minimizing CS artifacts, also contributed to decreasing both FN (true lesions masked by artifacts) and FP (signal voids due to CS artifact misinterpreted as lytic foci).

While most FP in the native ZTE sequence were caused by artifacts, those in BB and ZTE-DLCSC were primarily caused by benign lesions being misinterpreted as malignant. This is likely due to the limited definition of some subtle characteristic features of these lesions (*e.g*., thickened trabeculae in hemangiomas, peripheral sclerosis in Schmorl nodes or synovial cysts). This underlines the need for particular care when searching for these subtle features in these pseudo-CT sequences, as they may not display these features as clearly as CT scans do.

For several years, artificial intelligence-based solutions for image reconstruction have dramatically enhanced MRI by improving signal and image quality, reducing scan times, and facilitating visualization of anatomical structures and subtle lesions [33]. DL approaches, such as convolutional neural networks, are capable of reconstructing high-fidelity images from undersampled k-space data while preserving detail [17, 34]. Numerous anatomies have benefited from these innovations, including the brain, heart, and musculoskeletal system [35, 36].

Previous studies have illustrated the potential role of DL reconstructions to improve the value of ZTE sequences in various clinical applications, including spinal canal and neural foraminal stenosis, degenerative or traumatic shoulder or knee pathologies, inflammatory sacro-iliitis, and temporomandibular joint disorders [19, 20, 37, 38].

Several methods have been proposed to correct or cancel CS artifacts, such as increasing receiver bandwidth, using specialized pulse sequences (like short tau inversion-recovery or fat saturation), and advanced algorithms that combine data from multiple bandwidths, minimize CS artifacts and preserve image integrity. This technique reduces misregistration artifacts, enhances tissue contrast and eliminates signal voids that can obscure important pathology or can create pseudo-osteolytic lesions in the trabecular bone.

Regarding the count of osteolytic lesions, the three MRI sequences detected the same number of lesions in half of the cases. When a different number of lesions were detected, cases where ZTE-DLCSC detected more lesions were more frequent (+30% compared to ZTE; +20% compared to BB). CT detected more lytic lesions than MRI, though the difference in lesion count was minimal compared to ZTE-DLCSC. Additionally, CT exhibited better reproducibility and repeatability than pseudo-CT sequences. These observations suggest room for improvement in these sequences and quantify the scope of this improvement.

Our study has limitations. The patient cohort was small and monocentric. However, a large number of MM lesions were studied. The lumbar spine, pelvis and proximal femurs were covered to minimize the duration of MRI examinations, but are the most common sites of involvement by MM [23]. A CT obtained from a PET/CT scan was used. This limitation was overcome by prospectively optimizing the acquisition parameters of the CT to achieve a quality similar to that of an “oncological” low-dose whole-body CT obtained for MM in a radiology department. Finally, because we did not correlate observations from pseudo-CT sequences with anatomical and functional MRI sequences, we did not evaluate lesion activity or viability [39].

These limitations will be addressed in an ongoing study assessing the value of ZTE-DLCSC sequences covering the whole skeleton in a larger MM patient population and comparing their findings with CT and “marrow” MRI sequences.

In conclusion, this study shows that the implementation of the DL-CSC reconstruction algorithm into the ZTE sequence improves the repeatability and reproducibility of the readings and increases the diagnostic accuracy for the detection of osteolytic MM lesions compared to the native ZTE and BB sequences. By achieving high diagnostic accuracy for osteolytic lesions, the artificial intelligence-optimized ZTE sequence reinforces the perspective of a comprehensive, radiation-free MRI approach to bone marrow and mineral bone disease in MM.

## Supplementary information

**Additional File: Table S1**: Analysis testing the independence between regions and patients, for each reader and each MRI sequence, in terms of “error” using MRI compared to the consensus CT. **Table S2**: Causes of the false positive (FP) and false negative (FN) findings of pseudo-CT MRI sequences during readings by R1, R2 and R3, after comparison with the reference CT during the consensus adjudication.

## Data Availability

Data generated or analyzed during the study are available from the corresponding author by request.

## Abbreviations

^18^F-FDG-PET/CT: ^18^F-fluorodeoxyglucose-positron emission tomography/computed tomography
3D: Three-dimensional
AC1: Gwet’s agreement coefficient 1
AC2: Gwet’s agreement coefficient 2
BB: Black bone
CS: Chemical shift
CSC: Chemical shift correction
DL: Deep learning
FN: False negatives
FP: False positives
MM: Multiple myeloma
TN: True negatives
TP: True positives
ZTE: Zero echo time
ZTE-DLCSC: ZTE sequence reconstructed using DL and CSC
