# Supplementary material for: Zero echo time MRI with deep learning reconstruction and chemical shift correction for detecting osteolytic myeloma lesions
Source: Eur Radiol Exp. 2026 Jun 5;10:81. doi: 10.1186/s41747-026-00734-x (PMC13241374; doi:10.1186/s41747-026-00734-x)
Supplement: Supplementary file 1 — Additional File: Table S1: Analysis testing the independence between regions and patients, for each reader and each MRI sequence, in terms of “error” using MRI compared to the consensus CT. Table S2: Causes of the false positive (FP) and false negative (FN) findings of pseudo-CT MRI sequences during readings by R1, R2 and R3, after comparison with the reference CT during the consensus adjudication. [file 41747_2026_734_MOESM1_ESM.pdf]

Zero echo time MRI with deep learning reconstruction and chemical shift correction for detecting osteolytic myeloma lesions

ELECTRONIC SUPPLEMENTARY MATERIAL

Table S1: Analysis testing the independence between regions and patients, for each reader and each MRI sequence, in terms of “error” using MRI compared to the consensus CT. The test is based on the Goodman-Kruskal gamma for ordinal data [31] computed with Statsdirect software. Error was coded “1” when MRI and CT disagreed (i.e. when a false positive or a false negative is yielded using MRI) and coded “0” when both techniques agreed. All tests but one are non-significant ($p > 0.05$), showing that there is little evidence of an association between regions and patients in terms of error, regardless of the reader or the sequence. The significant association is observed with senior reader R1 using ZTE-DLCSC ($p = 0.024$), that may be explained by the fact that few error occurred using this sequence, but when errors occurred, they were more frequent in the right femur (3 errors over 10 patients); an observation that is not confirmed in the left femur where no error is observed, suggesting that the association observed was fortuitous. 95% confidence intervals are given into brackets.

		Goodman-Kruskal gamma	*p*-value
	**ZTE**	-0.05 [-0.28; 0.18]	0.654
**R1**	**ZTE-DLCSC**	-0.54 [-1.01; -0.07]	0.024*
	**BB**	-0.18 [-0.40; 0.04]	0.101
	**ZTE**	-0.01 [-0.24; 0.22]	0.922
**R2**	**ZTE-DLCSC**	-0.21 [-0.64; 0.19]	0.352
	**BB**	-0.05 [-0.29; 0.19]	0.691
	**ZTE**	0.04 [-0.22; 0.31]	0.742
**R3**	**ZTE-DLCSC**	-0.07 [-0.49; 0.34]	0.736
	**BB**	-0.11 [-0.43; 0.20]	0.480

Table S2: Causes of the false positive (FP) and false negative (FN) findings of pseudo-CT MRI sequences during readings by R1, R2 and R3, after comparison with the reference CT during the consensus adjudication.

Percentages are computed from R1, R2 and R3 (1st reading only). FN are either undetected lesions (visible but not seen by the reader); undetectable lesions (not visible due to the technical limitation/insufficient signal); or true myeloma lesions considered as benign (hemangiomas, cysts) or Chemical Shift (CS) artifacts. FP are either benign conditions (Schmorl nodes, subchondral cysts, herniation pits, hemangiomas, veinous dilatations) considered as malignant; CS artifacts creating pseudo-lesions; or true lytic myeloma lesions "missed" on the CT considered as reference. The upper part of the Table illustrates FN for all Readers; the lower part illustrates FP. Table reads as follows, for column R1: amongst his 100 readings (10 patients x 10 regions), R1 had 25 FN using ZTE, 2 FN using ZTE-DLCSC and 17 FN using BB; amongst these 100 readings, R1 had 14 FP using ZTE, 5 FP using ZTE-DLCSC and 9 FP using BB.

Overall, the following observations are made. Among the three MRI sequences, ZTE had the most FN (73) and FP (44), as well as a higher FN-to-FP ratio (**Fig. 3**). In comparison, BB had 32% fewer FN and 23% fewer FP than native ZTE. ZTE-DLCSC had even fewer FN (75% fewer than ZTE and 64% fewer than BB) and FP (45% fewer than ZTE and 29% fewer than BB).

Most of the false negative (FN) observations of ZTE corresponded to undetectable lesions due to technical limitations (62%), i.e. low SNR of the sequence **(Fig. 4)**. The second cause of FN corresponded to lesions missed by the readers (27%). In comparison, FN observations from ZTE-DLCSC corresponded to lesions missed by the reader (67%) rather than to technical limitations (28%, i.e. almost three times less than ZTE). Similarly, to ZTE-DLCSC, FN of BB corresponded to missed lesions (48%) rather than to technical limitations (24%). A small number of FN resulted from true MM lesions misinterpreted as benign conditions or CS artifacts (ZTE: 11%; ZTE-DLCSC: 5.6%; BB: 28%).

The FP observations of ZTE were due equally to benign lesions that were misinterpreted as malignant (41%) and to CS artifacts that were misinterpreted as lytic lesions (43%). The latter cause of FP was minimal with ZTE-DLCSC (8.3%) and BB (0.0%), where the main cause of FP was benign lesions considered malignant (63% with ZTE-DLCSC and 74% with BB). Additionally, there were a few false-positive readings of pseudo-CT MRI sequences that represented missed lesions on the reference CT. These false-positive readings were

confirmed to be true lesions of pseudo-CT sequences during the consensus reading of all available MRI sequences: DWI, T1, and fat sequences (ZTE: 14%; ZTE-DLCSC: 29%; BB: 26%) **(Fig. 5)**.

		R1<br>Senior	R2<br>Junior	R3<br>Junior	R2 (2nd)<br>Junior	R1, R2, R3 (1st reading of R2)
**FN findings**	**Causes**	**N**	**N**	**N**	**N**	Total
**ZTE**	**Total FN/ Total regions**	**25/100**	**26/100**	**22/100**	**30/100**	**73**
	Undetected (missed by reader)	7	7	6	8	20 (27%)
	Undetectable (technical limitation)	16	15	14	17	45 (62%)
	Lesion considered as mechanical/artifact	2	4	2	5	8 (11%)
**ZTE-DLCSC**	**Total FN/ Total regions**	**2/100**	**9/100**	**7/100**	**14/100**	**18**
	Undetected (missed by reader)	1	6	5	7	12 (67%)
	Undetectable (technical limitation)	1	2	2	2	5 (28%)
	Lesion considered as mechanical/artifact	0	1	0	5	1 (5.6%)
**BB**	**Total FN/ Total regions**	**17/100**	**22/100**	**11/100**	**20/100**	**50**
	Undetected (missed by reader)	9	10	5	9	24 (48%)
	Undetectable (technical limitation)	4	5	3	5	12 (24%)
	Lesion considered as mechanical/artifact	4	7	3	6	14 (28%)
**FP findings**	**Causes**	**N**	**N**	**N**	**N**	Total (%)
**ZTE**	**Total FP/ Total regions**	**14/100**	**17/100**	**13/100**	**19/100**	**44**
	Benign considered as malignant	6	7	6	7	18 (41%)
	Missed on CT (true positive)	2	2	2	2	6 (14%)
	Pseudo-lytic lesions due to CS artifacts	6	8	5	10	19 (43%)
**ZTE-DLCSC**	**Total FP/ Total regions**	**5/100**	**11/100**	**8/100**	**9/100**	**24**
	Benign considered as malignant	3	7	5	5	15 (63%)
	Missed on CT (true positive)	2	3	2	2	7 (29%)
	Pseudo-lytic lesions due to CS artifacts	0	1	1	2	2 (8.3%)
**BB**	**Total FP/ Total regions**	**9/100**	**12/100**	**13/100**	**17/100**	**34**
	Benign considered as malignant	6	9	10	11	25 (74%)
	Missed on CT (true positive)	3	3	3	5	9 (26%)
	Pseudo-lytic lesions due to CS artifacts	0	0	0	1	0 (0.0%)

BB Black bone pseudo-CT sequence, *CT* Computed tomography, *ZTE* Zero echo time pseudo-CT sequence, *ZTE-DLCSC* ZTE sequence reconstructed with deep learning and chemical shift correction algorithm.
